# Comparison of ^±^σ-hole and ^±^R˙-hole interactions formed by tetrel-containing complexes: a computational study†

**DOI:** 10.1039/d0ra09564h

**Published:** 2021-01-19

**Authors:** Mahmoud A. A. Ibrahim, Ebtisam M. Z. Telb

**Affiliations:** Computational Chemistry Laboratory, Chemistry Department, Faculty of Science, Minia University Minia 61519 Egypt m.ibrahim@compchem.net

## Abstract

For the first time, unconventional ^±^R˙-hole interactions were unveiled in tetrel-containing complexes. The nature and characteristics of ^±^R˙-hole interactions were explored relative to their ^±^σ-hole counterparts for ˙TF_3_⋯ and W–T–F_3_⋯B/R˙/A complexes (where T = C, Si, and Ge, W = H and F, B = Lewis bases, R˙ = free radicals, and A = Lewis acids). In an effort to thoroughly investigate such interactions, a plethora of quantum mechanical calculations, including molecular electrostatic potential (MEP), maximum positive electrostatic potential (*V*_s,max_), point-of-charge (PoC), interaction energy, symmetry adapted perturbation theory (SAPT), and reduced density gradient–noncovalent interaction (RDG–NCI) calculations, were applied. The most notable findings to emerge from this study are that (i) from the electrostatic perspective, the molecular stabilization energies of ˙TF_3_ and W–T–F_3_ monomers became more negative as the Lewis basicity increased, (ii) the most stable complexes were observed for the ones containing Lewis bases, forming ^−^σ-hole and ^−^R˙-hole interactions, and the interaction energies systematically increased in the order H–T–F_3_⋯B < ˙TF_3_⋯B < F–T–F_3_⋯B, (iii) contrariwise, the ^+^σ-hole and ^+^R˙-hole interactions with Lewis acids are more energetically favorable in the order F–T–F_3_⋯A < ˙TF_3_⋯A < H–T–F_3_⋯A, and (iv) generally, the dispersion force plays a key role in stabilizing the tetrel-containing complexes, jointly with the electrostatic and induction forces for the interactions with Lewis bases and acids, respectively. Concretely, the findings presented in this paper add to our understanding of the characteristics and nature of such intriguing interactions.

## Abstract 2

The characteristics and nature of ^±^R˙-hole interactions were uncovered for the first time and compared to their ^±^σ-hole analogs in ˙TF_3_⋯ and W–T–F_3_⋯B/R˙/A complexes.

## Graphical abstract

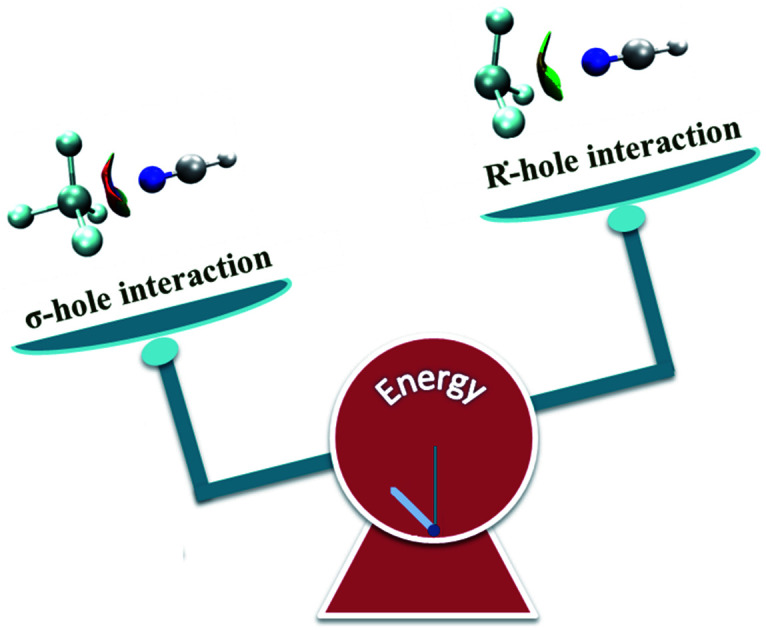

## 1. Introduction

σ-Hole interaction is a major area of interest within the realm of noncovalent interactions owing to its primordial roles in molecular recognition,^1,2^ crystal materials,^3,4^ and biological systems.^5,6^ Accordingly, researchers have focused more attention on its characteristics and nature which have been widely studied from both computational and experimental aspects.^7–11^ These studies collectively proclaimed a series of different types of σ-hole-based interactions—namely, tetrel^12,13^ pnicogen^14,15^ chalcogen^16,17^ halogen^18,19^ and aerogen bonds^20,21^ embracing the Group IV–VIII elements, respectively. The σ-hole interactions can be traced back to the presence of a positive or less negative region along the extension of the covalently bonded Group IV–VIII elements (termed a σ-hole).^22–24^

Among the σ-hole interactions, tetrel bonding has received immense interest due to its technological and fundamental magnitude in supramolecular chemistry^25^ and dynamical processes such as protein folding and ligand–acceptor interactions.^26,27^ Several studies have documented the potentiality of the tetrel-containing molecules to form ^−^σ-hole interactions as a consequence of the interaction of Group IV elements (T) *via* the σ-hole with Lewis bases (B) such as lone-pair (lp),^28–31^ anion,^32,33^ π-systems.^34,35^ Recently, a thought-provoking study has given prominence to ^+^σ-hole interactions of tetrel-containing complexes, which demonstrated the ability of tetrel-containing molecules to preferentially interact with Lewis acids (A).^30^ Nonetheless, only a handful of reports in the literature were introduced to investigate the intermolecular interactions of tetrel-containing molecules with Lewis acids.

In light of recent research, single-electron noncovalent interactions were reported, referring to an interaction between the unpaired electron of a radical (R˙) acting as a Lewis base and the σ-hole on the Group IV–VIII elements acting as a Lewis acid.^36–44^ Considering the pivotal role of radicals in combustion, polymerization, biochemistry, and many other chemical processes,^45–48^ further studies are needed to characterize the interactions of radical species with tetrel-containing molecules.

One of the most significant current investigations in noncovalent interactions is that ascribed to the lp-hole. Lp-hole interactions were accentuated for Group V–VIII elements as a result of a positive region opposite to the lp (called lp-hole).^49–51^ According to the term lp-hole, it is not plausible for sp^3^-hybridized tetrel-containing molecules to form this type of interaction. A question has been raised about whether sp^3^-hybridized tetrel radicals (˙TX_3_) can form another intriguing interaction so-called R˙-hole interaction. This interaction is attributed to the presence of a positive region opposite to the unpaired electron on the tetrel (termed, R˙-hole). As far as our knowledge extends, no previous study has assessed the occurrence of R˙-hole interaction.

Subsequently, the objectives of the current work were to (i) uncover the occurrence of the R˙-hole interactions and (ii) gain insight into the characteristics and nature of the σ-hole and R˙-hole interactions of tetrel-containing molecules with Lewis bases, free radicals, and Lewis acids. ^−^R˙-hole and ^+^R˙-hole interactions were assigned for the interactions with Lewis bases and acids, by analogy with ^−^σ-hole and ^+^σ-hole interactions, respectively. The point-of-charge (PoC) approach was utilized to precisely determine the R˙-hole location and elucidate the ^±^σ-hole and ^±^R˙-hole interactions from the electrostatic perspective.^30,49,52–56^ The interaction energies for ˙TF_3_⋯ and W–T–F_3_⋯B/R˙/A complexes (where T = C, Si, and Ge, and W = H and F) were calculated at the MP2/aug-cc-pVTZ(PP) and CCSD(T)/CBS levels of theory. The symmetry adapted perturbation theory (SAPT) and reduced density gradient–noncovalent interaction (RDG–NCI) index calculations were executed to reconnoiter the characteristics and nature of such interactions. This study makes a major contribution to research on tetrel radicals-bonded complexes by demonstrating the characteristics and nature of R˙-hole interactions compared to the σ-hole ones for the first time.

## 2. Computational methods

In the current study, ˙TF_3_ and W–T–F_3_ model systems were chosen as a case study to investigate the σ-hole and R˙-hole interactions with Lewis bases, acids, and free radicals (where T = C, Si, and Ge, and W = H and F). NCH and FH molecules were investigated as Lewis bases and acids, while free radicals were exemplified by ˙CH_3_ and ˙CF_3_. The geometries of the systems were initially optimized at the MP2/aug-cc-pVTZ level of theory, with treating Ge atom with aug-cc-pVTZ-PP basis set to account for relativistic effects.^57–59^ The molecular electrostatic potential (MEP) maps were generated for the optimized monomers and then mapped on 0.002 au electron density contours based on the previous recommendations.^24,60^ Moreover, the maximum positive electrostatic potential (*V*_s,max_) calculations at σ-hole and R˙-hole locations were carried out using the Multiwfn3.7 software.^61^

**Fig. 1:**
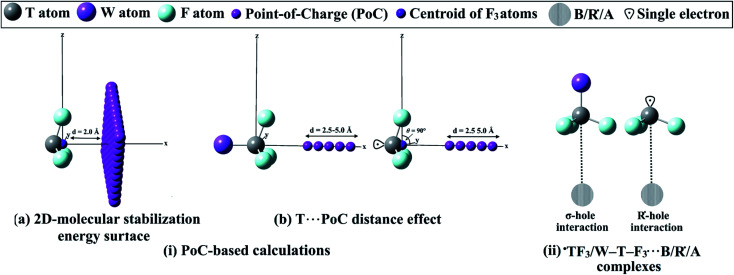
Schematic representation of (i) implemented PoC-based calculations and (ii) ˙TF_3_⋯ and W–T–F3⋯B/R˙/A complexes.

To fulfill the aim of this investigation, the R˙-hole location was first precisely determined. The studied ˙TF_3_ radicals were aligned to the *x*-axis, and the *yz* plane was then scanned by −0.01 au PoC with a step size of 0.1 Å, using the point-of-charge (PoC) approach (Fig. 1(ia)). The PoC was placed at a distance of 2.0 Å from the tetrel atom and moved along both *y*- and *z*-directions in a range from 1.6 to −1.6 Å, generating 2D-molecular stabilization energy surfaces (see Fig. 1(ia)). To inspect the effect of Lewis basicity and acidity on the ^±^σ-hole and ^±^R˙-hole interactions strength, different values of negatively and positively charged points stimulating Lewis bases and acids, respectively, were applied. In the latter calculation, the studied monomers were aligned to the *x*-axis, and the T⋯PoC distance effect on the strength of interaction was evaluated in the range 2.5 to 5.0 Å (see Fig. 1(ib)). The employed PoC values were set to ±0.10, ±0.25, ±0.50, and ±1.00 au. The strength of the ^±^σ-hole and ^±^R˙-hole interactions was estimated at MP2/aug-cc-pVTZ(PP) level of theory in terms of the molecular stabilization energy (*E*_stablization_) according to the following equation:^30,49,50,52–56^*E*_stablization_ = *E*_tetrel-containing molecule⋯PoC_ − *E*_tetrel-containing molecule_

To analyze the potentiality of ˙TF_3_ and W–T–F_3_ systems to favorably interact *via* the σ-hole and R˙-hole with B, R˙, and A (see Fig. 1(ii)), full geometrical optimization of the complexes in *C*_3V_ space group was performed at the MP2/aug-cc-pVTZ level of theory (with PP functions for Ge atom). Vibrational frequency calculations were not performed for the binary complexes; thus, there was a possibility that the structures were not energetic minima. The interaction energies of the binary complexes were then estimated at the same level of optimization and performed with taking basis set superposition error (BSSE) correction into account.^62^ The interaction energies were computed as the difference in energy between the complex and the sum of monomers with the same geometries as in the complex. The frozen-core (FC) approximation was adopted for all MP2 calculations. Furthermore, the interaction energies were calculated for the optimized complexes at the CCSD(T)/CBS level of theory. The CCSD(T)/CBS energies were calculated based on the following equation:^63^*E*_CCSD(T)/CBS_ = Δ*E*_MP2/CBS_ + Δ*E*_CCSD(T)_where:Δ*E*_MP2/CBS_ = (64*E*_MP2/aug-cc-pVQZ_ − 27*E*_MP2/aug-cc-pVTZ_)/37Δ*E*_CCSD(T)_ = *E*_CCSD(T)/aug-cc-pVdZ_ − *E*_MP2/aug-cc-pVDZ_

To have a more in-depth insight into the nature of σ-hole and R˙-hole interactions, the energy decomposition calculations were performed through the symmetry adapted perturbation theory-based energy decomposition analysis (SAPT-EDA) method using PSI4 code.^64,65^ The SAPT-EDA method provides a separation of interaction energies into four physically meaningful components, such as those arising from electrostatics (*E*_elst_), exchange (*E*_exch_), induction (*E*_ind_), and dispersion (*E*_disp_). The interaction energies were estimated at SAPT0 truncation along with an aug-cc-pVTZ basis set, given as follows:^66–68^*E*^SAPT0^_int_ = *E*_elst_ + *E*_exch_ + *E*_ind_ + *E*_disp_where:*E*_elst_ = *E*_elst_^(10)^*E*_exch_ = *E*_exch_^(10)^*E*_ind_ = *E*_ind,resp_^(20)^ + *E*_exch-ind,resp_^(20)^ + δ*E*_HF_^(2)^*E*_disp_ = *E*_disp_^(20)^ + *E*_exch-disp_^(20)^

To analyze the bonding characteristics of σ-hole and R˙-hole interactions, reduced density gradient–noncovalent interaction (RDG–NCI) indices were constructed, and the NCI plots were also illustrated.^69^ The gradient isosurfaces were colored on a blue-green-red (BGR) scale, where blue surfaces refer to strong attractions, green surfaces show weak interactions, and red surfaces imply strong repulsions. The coloring scale of electron density (*ρ*) was from −0.035 (blue) to 0.020 (red) au. The RDG–NCI analysis was carried out using Multiwfn3.7 software and then visualized by VMD1.9.2 software.^70^ The geometrical optimization, MEP analysis, interaction energy, and PoC-based calculations were performed using Gaussian09 software.^71^

## 3. Results and discussion

### 3.1. MEP and *V*_s,max_ calculations

**Fig. 2:**
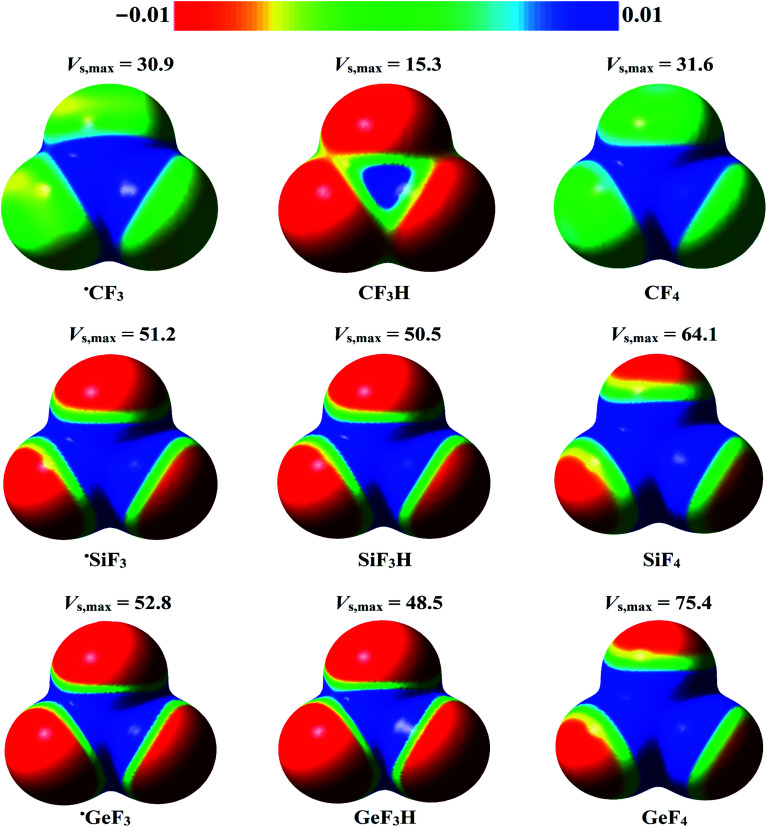
Molecular electrostatic potential (MEP) maps of the investigated ˙TF_3_ and W–T–F_3_ systems (where T = C, Si, and Ge, and W = H and F) plotted at 0.002 au electron density contours. The electrostatic potentials vary from −0.01 au (red) to 0.01 au (blue). The calculated maximum positive electrostatic potential (*V*_s,max_, kcal mol^−1^) at the σ-hole and R˙-hole are also depicted.

The molecular electrostatic potential (MEP) model is widely used for deducing the reactive sites on isolated monomers that can, in turn, provide potential sites for electrophilic or nucleophilic attacks.^72^ Therefore, the MEP maps were generated at MP2/aug-cc-pVTZ (PP) level of theory for the studied monomers to ascertain the occurrence of σ-hole and R˙-hole regions. The *V*_s,max_ values at the σ-hole, and R˙-hole were also estimated using Multiwfn3.5 software. The generated MEP maps and the estimated *V*_s,max_ values for the studied monomers are illustrated in Fig. 2.

According to the data presented in Fig. 2, the studied tetrel-containing molecules exhibited variable-in-size σ-holes along the W–T bond extensions (where T = C, Si, and Ge, and W = H and F). The σ-hole size was increased with moving from lighter to heavier tetrel atoms in order C < Si < Ge. For example, the *V*_s,max_ values at the σ-hole were found to be 31.6, 64.1, and 75.4 kcal mol^−1^ for F–C–F_3_, F–Si–F_3_, and F–Ge–F_3_ molecules, respectively. As well, a direct correlation between the electronegativity of the W atom and σ-hole size of the tetrel atom was observed. Taking W–Ge–F_3_ system as an example, the obtained *V*_s,max_ values at the σ-hole for H–Ge–F_3,_ and F–Ge–F_3_ were 48.5 and 75.4 kcal mol^−1^, respectively.

Interestingly, a positive region opposite to the unpaired electron (*i.e.*, R˙-hole) was noticed in the studied radicals. The *V*_s,max_ magnitude at the R˙-hole attenuated in the order ˙GeF_3_ > ˙SiF_3_ > ˙CF_3_ and obtained with values of 52.8, 51.2, and 30.9 kcal mol^−1^, respectively. Generally, the *V*_s,max_ values at the σ-hole were higher than those at R˙-hole for the same monomer.

### 3.2. PoC-based calculations

#### 3.2.1. R˙-hole location

**Fig. 3:**
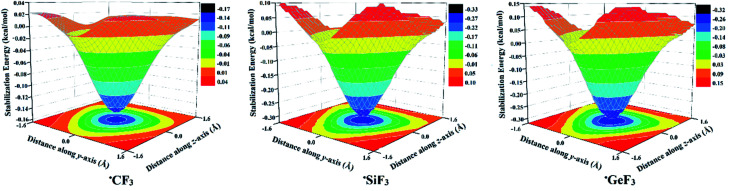
Generated 2D-molecular stabilization energy surfaces for the investigated ˙CF_3_, ˙SiF_3_, and ˙GeF_3_ monomers in the presence of −0.01 PoC at a T⋯PoC distance of 2.0 Å along the *x*-axis (see Computational methods section for details).

To achieve the aim of the study, the optimum R˙-hole location for the studied ˙TF_3_ radicals remained to be precisely determined. Based on a previous recommendation, *V*_s,max_ calculations are unreliable in determining the location of the maximum positive electrostatic potential.^52^ Therefore, the 2D-molecular stabilization energy surfaces were generated with the help of the PoC approach and depicted in Fig. 3.

As shown in Fig. 3, the largest molecular stabilization energies (*i.e.*, more negative) were observed at the centroid of the F_3_ atoms with values of −0.15, −0.30, and −0.31 kcal mol^−1^ for ˙CF_3_, ˙SiF_3_, and ˙GeF_3_ radicals, respectively. These findings are in line with those for lp-hole containing molecules, where the precise lp-hole positions were identified at the centroid of the *xyz* plane in the covalently bonded Group V-VIII elements.^49^

#### 3.2.2. Lewis basicity and acidity effects

**Fig. 4:**
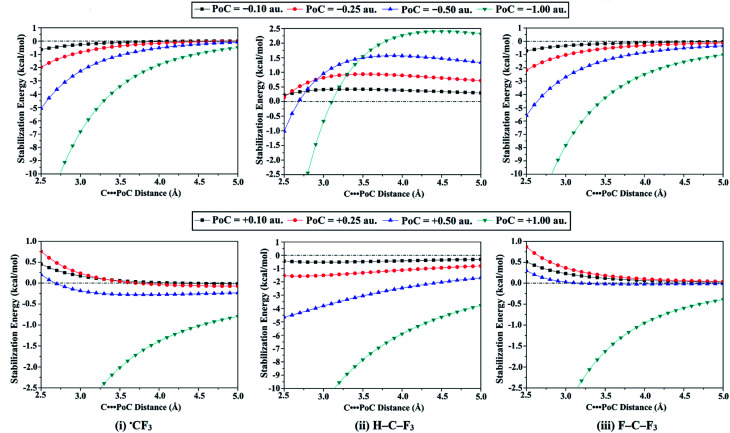
Generated molecular stabilization energy curves for the (i) ˙CF_3_, (ii) H–C–F_3_, and (iii) F–C–F_3_ monomers in the presence of ±0.10, ±0.25, ±0.50, and ±1.00 au PoCs at T⋯PoC distances ranging from 2.5 Å to 5.0 Å.

Towards scrutinizing the ^±^σ-hole and ^±^R˙-hole interactions from the electrostatic perspective, the Lewis basicity and acidity effects were elucidated with the incorporation of the PoC approach. Thence, the molecular stabilization energy curves for ˙TF_3_ and W–T–F_3_ monomers were generated at T⋯PoC distance in a range 2.5 to 5.0 Å along the σ-hole and R˙-hole extensions (see Fig. S1 and S2†). The molecular stabilization energy curves of carbon-containing molecules were taken as an example and compared in Fig. 4. Table 1 lists the values of molecular stabilization energies at T⋯PoC distance of 2.5 Å for the investigated monomers.

The molecular stabilization energies (in kcal mol^−1^) of the studied ˙TF_3_ and W–T–F_3_ systems (where T = C, Si, and Ge, and W = H and F) in the presence of ±0.10, ±0.25, ±0.50, or ±1.00 au PoCs at a T⋯PoC distance of 2.5 Å

**Table:** 

Molecule	Molecular stabilization energies (kcal mol^−1^) at 2.5 Å			
	PoC = −0.10	PoC = −0.25	PoC = −0.50	PoC = −1.00
˙CF_3_	−0.65	−1.96	−5.06	−14.50
H–C–F_3_	0.22	0.14	−1.01	−6.94
F–C–F_3_	−0.72	−2.18	−5.58	−15.82
˙SiF_3_	−1.19	−3.47	−8.51	−22.96
H–Si–F_3_	−1.10	−3.24	−8.03	−21.91
F–Si–F_3_	−1.84	−5.06	−11.57	−28.62
˙GeF_3_	−0.20	−3.01	−7.73	−21.92
H–Ge–F_3_	−0.76	−2.44	−6.61	−19.71
F–Ge–F_3_	−2.21	−6.00	−13.55	−32.91

**Table:** 

Molecule	Molecular stabilization energies (kcal mol^−1^) at 2.5 Å			
	PoC = +0.10	PoC = +0.25	PoC = +0.50	PoC = +1.00
˙CF_3_	0.45	0.76	0.20	−5.75
H–C–F_3_	−0.44	−1.54	−4.64	−16.81
F–C–F_3_	0.51	0.87	0.30	−6.07
˙SiF_3_	0.92	1.77	1.62	−1.19
H–Si–F_3_	0.83	1.53	1.12	−7.63
F–Si–F_3_	1.59	3.46	5.12	1.23
˙GeF_3_	0.69	1.14	0.17	−10.22
H–Ge–F_3_	0.46	0.55	−1.04	−12.88
F–Ge–F_3_	1.93	4.30	6.67	3.74

Concerning ^−^σ-hole and ^−^R˙-hole interactions, the molecular stabilization energies were boosted by increasing the negativity of PoCs (*i.e.*, Lewis basicity) and decreasing the T⋯PoC distances. For example, the observed molecular stabilization energies for ˙CF_3_ monomer were −0.65, −1.96, −5.06, and −14.5 kcal mol^−1^ at T⋯PoC distance of 2.5 Å in the presence of −0.10, −0.25, −0.50, and −1.00 au PoCs, respectively (see Table 1). Furthermore, the effect of Lewis basicity was most distinguishable for the F–T–F_3_ followed in order by the ˙TF_3_ and H–T–F_3_ systems. For instance, the obtained molecular stabilization energies at T⋯PoC distance of 2.5 Å for F–Ge–F_3_, ˙GeF_3_, and H–Ge–F_3_ monomers were −32.91, −21.92, and −19.71 kcal mol^−1^ in case of −1.00 au PoC, respectively. The observation can explain this pattern that the systematic growth of the *V*_s,max_ magnitude at σ-hole, and R˙-hole led to a gradual increase in the molecular stabilization energies (*i.e.*, became more negative). More interestingly, according to Fig. 4, molecular stabilization energies remained to be observed at long T⋯PoC distances for ˙CF_3_ and F–C–F_3_ molecules, demonstrating the dominance of the attractive electrostatic force between the negative PoC and the positive electrophilic sites over the tetrel atom (*i.e.*, σ-hole and R˙-hole). Otherwise, in the case of H–C–F_3_ molecule, molecular destabilization energies were noticed and augmented by increasing the Lewis basicity, indicating the repulsive electrostatic interaction with F_3_ atoms is the controlling one at long T⋯PoC distance.

For ^+^σ-hole and ^+^R˙-hole interactions, molecular stabilization energies were observed for all investigated molecules, except SiF_4_ and GeF_4_. The incompetence of SiF_4_ and GeF_4_ to participate in ^+^σ-hole interaction is appertaining to the significant repulsive interaction between the positive PoC and the excessive positive σ-hole. For H–C–F_3_ monomer as an exemplar, H–C–F_3_ demonstrated molecular stabilization energies of −0.44, −1.54, −4.64, and −16.81 kcal mol^−1^ at T⋯PoC distance of 2.5 Å in the presence of +0.10, +0.25, +0.50, and +1.00 au PoCs, respectively. As claimed by the latter observation, the molecular stabilization energies boosted with increasing the positivity of the PoC (*i.e.*, Lewis acidity) despite the repulsive electrostatic interaction between the positive σ-hole and positive PoC, illustrating the effectual role of polarization in stabilizing such interactions. For some tetrel-containing molecules, generally, unfavorable electrostatic interactions were noticed at very short T⋯PoC distances. It is apparent from Table 1 that the H–T–F_3_ system gave more favorable electrostatic interaction compared to ˙TF_3_ and F–T–F_3_ systems. For instance, the molecular stabilization energies were found to increase in the order of F–C–F_3_ < ˙CF_3_ < H–C–F_3_ with values of −5.75, −6.07, and −16.81 kcal mol^−1^ at 2.5 Å in the presence of +1.00 au PoC, respectively.

Overall, with respect to the research question, it was found that the studied monomers can electrostatically interact with Lewis bases and acids along the R˙-hole extension, forming ^±^R˙-hole interactions. Compared with the ^−^σ-hole and –R˙-hole interactions, the parallel ^+^σ-hole and ^+^R˙-hole interactions with Lewis acids are less favorable. The substantial role of polarization effect and F_3_ atoms in forming such interactions cannot be neglected.

### 3.3. Tetrel⋯B/R˙/A complexes

#### 3.3.1. Interaction energy

To affirm the potency of the studied monomers to participate in ^±^σ-hole and ^±^R˙-hole interactions, geometrical optimization was performed for ˙TF_3_⋯ and W–T–F_3_⋯B/R˙/A complexes. The interaction energies were then estimated at MP2/aug-cc-pVTZ(PP) and CCSD(T)/CBS levels of theory (see Table 2).

Interaction energies (in kcal mol^−1^) calculated at MP2/aug-cc-pVTZ(PP) and CCSD(T)/CBS levels of theory for the optimized ˙TF_3_⋯ and W–T–F3⋯B/R˙/A complexes (where T = C, Si, and Ge, W = H and F, B = Lewis bases, R˙ = free radicals, and A = Lewis acids), and T⋯ B/R˙/A intermolecular distances (*d*, Å)

**Table:** 

Complex	˙TF_3_/W–T–F_3_⋯B systems					
	NCH			FH		
	*E*_MP2_	*E*_CCSD(T)_	*d*_T⋯NCH_	*E*_MP2_	*E*_CCSD(T)_	*d*_T⋯FH_
˙CF_3_⋯B	−1.04	−1.14	3.29	−0.58	−0.75	3.18
H–C–F_3_⋯B	0.01	−0.04	3.55	0.08	−0.03	3.39
F–C–F_3_⋯B	−1.09	−1.15	3.36	−0.62	−0.79	3.23
˙SiF_3_⋯B	−2.08	−2.28	3.17	−0.77	−1.03	3.23
H–Si–F_3_⋯B	−1.95	−2.10	3.18	−0.70	−0.95	3.22
F–Si–F_3_⋯B	−3.44	−3.64	3.01	−1.37	−1.67	3.10
˙GeF_3_⋯B	−2.70	−2.95	2.96	−0.56	−0.89	3.14
H–Ge–F_3_⋯B	−2.13	−2.32	3.01	−0.39	−0.67	3.21
F–Ge–F_3_⋯B	−10.21	−17.83	2.39	−1.84	−2.29	2.95

a Optimized structure could not be obtained.

**Table:** 

Complex	˙TF_3_/W–T–F_3_⋯R˙ systems					
	˙CH_3_			˙CF_3_		
	*E*_MP2_	*E*_CCSD(T)_	*d*_T···˙CH_3__	*E*_MP2_	*E*_CCSD(T)_	*d*_T···˙CF_3__
˙CF_3_⋯R˙	−0.41	−0.57	3.74	−0.27	−0.47	3.55
H–C–F_3_⋯R˙	−0.26	−0.43	3.86	−0.56	−0.78	3.52
F–C–F_3_⋯R˙	−0.46	−0.63	3.75	−0.34	−0.50	3.53
˙SiF_3_⋯R˙	−0.69	−0.98	3.65	−0.37	−0.65	3.58
H–Si–F_3_···R˙	−0.72	−1.02	3.61	−0.42	−0.73	3.52
F–Si–F_3_···R˙	−1.03	−1.40	3.47	−0.25	−0.54	3.47
˙GeF_3_⋯R˙	−0.74	−1.19	3.47	−0.35	−0.85	3.36
H–Ge–F_3_⋯R˙	−0.70	−1.11	3.52	−0.51	−0.97	3.39
F–Ge–F_3_⋯R˙	−1.79	−2.42	3.17	−0.19	−0.64	3.25

**Table:** 

Complex	˙TF_3_/W–T–F_3_⋯A systems					
	HCN			HF		
	*E*_MP2_	*E*_CCSD(T)_	*d*_T⋯HCN_	*E*_MP2_	*E*_CCSD(T)_	*d*_T⋯HF_
˙CF_3_⋯A	0.07	0.02	2.96	0.11	0.09	3.49
H–C–F_3_⋯A	−1.07	−1.14	2.88	−0.79	−0.88	2.91
F–C–F_3_⋯A	0.00	−0.03	2.99	0.15	0.14	3.26
˙SiF_3_⋯A	−0.14	−0.16	3.41	0.07	0.04	3.97
H–Si–F_3_⋯A	−0.18	−0.22	3.53	−0.08	−0.09	4.62
F–Si–F_3_⋯A	0.49	0.48	3.55	—a	—a	—a
˙GeF_3_⋯A	−0.34	−0.46	3.14	−0.01	−0.07	3.46
H–Ge–F_3_⋯A	−0.58	−0.72	3.10	−0.28	−0.34	3.57
F–Ge–F_3_⋯A	0.44	0.43	3.86	—a	—a	—a

As can be readily appreciated from Table 2, the investigated monomers exhibited a strong tendency to interact with Lewis bases, Lewis acids, and free radicals with CCSD(T)/CBS interaction energies in the range −0.03 to −17.83, 0.48 to −0.72, and −0.43 to −2.42 kcal mol^−1^, respectively. For the analyzed complexes, the interaction energies were almost greater (*i.e.*, more negative) for the Ge-containing complexes compared to the Si and C counterparts, which are in agreement with the MEP results (see Fig. 2). Noteworthy, reasonable differences between the interaction energies estimated at MP2/aug-cc-pVTZ(PP) and those calculated at CCSD(T)/CBS were observed, demonstrating the accuracy and effectiveness of the MP2/aug-cc-pVTZ(PP) level of theory to investigate such noncovlanet interactions. This is in agreement with previous studies on estimating the strength of noncovlent interactions in related systems^28,32,73–76^ (Table 2).

In regards to ˙TF_3_⋯ and W–T–F_3_⋯B complexes, ^−^σ-hole, and ^−^R˙-hole interaction energies were large negative values, indicating strong interactions between the interacting subunits. A linear correlation was found between the interaction energies and the *V*_s,max_ at the σ-hole and R˙-hole over the tetrel atom, where the interaction energies enhanced in order H–T–F_3_⋯B < ˙TF_3_⋯B < F–T–F_3_⋯B. For example, the CCSD(T)/CBS interaction energies for H–Ge–F_3_⋯, ˙GeF_3_⋯, and F–Ge–F_3_⋯NCH complexes were noticed with values of −2.32, −2.95, and −17.83 kcal mol^−1^, respectively. It was also found that the greater the atomic size of tetrel atom, the shorter T⋯B contact, which in turn resulted in strengthening interaction energies. The example given was for F–C–F_3_⋯, F–Si–F_3_⋯, and F–Ge–F_3_⋯NCH complexes, where the CCSD(T)/CBS interaction energies were noticed with values of −1.15, −3.64, and −17.83 kcal mol^−1^ at T⋯N intermolecular distances values of 3.36, 3.01, and 2.39 Å, respectively (see Table 2). Moreover, the NCH Lewis base was found to interact more strongly than the FH candidate with the studied monomers.

For ˙TF_3_⋯ and W–T–F_3_⋯A complexes, favorable ^+^σ-hole and ^+^R˙-hole interactions were observed for most of the studied complexes. The inability of some monomers to engage in favorable interactions with Lewis acids can be attributed to the dominance of the repulsive electrostatic interaction between the electrophilic site over the tetrel atom and the Lewis acid. Generally, the interaction energies reinforced by decreasing the *V*_s,max_ value over the tetrel atom in the order F–T–F_3_⋯A < ˙TF_3_⋯A < H–T–F_3_⋯A. As an exemplar, the CCSD(T)/CBS interaction energies were found with values of 0.43, −0.46, and −0.72 kcal mol^−1^ for F–Ge–F_3_⋯, ˙GeF_3_⋯, and H–Ge–F_3_⋯HCN complexes, respectively. However, the CCSD(T)/CBS interaction energies for ˙CF_3_⋯, ˙SiF_3_⋯ and ˙GeF_3_⋯HCN complexes were with values of 0.02, −0.16, and −0.46 kcal mol^−1^, respectively, despite increasing the *V*_s,max_ at R˙-hole in order of C < Si < Ge. These findings explicitly referred to the interaction of F_3_ atoms with the H atom of the Lewis acid and were further investigated by NCI analysis. Compared to HF-containing complexes, the interaction energies for HCN-containing complexes were systematically larger (*i.e.*, more negative). For example, the CCSD(T)/CBS interaction energies for H–Ge–F_3_⋯HCN and ⋯HF complexes were −0.72 and −0.34 kcal mol^−1^, respectively.

In the case of ˙TF_3_⋯ and W–T–F_3_⋯R˙ complexes, which exhibited a staggered conformation with C_3_v symmetry, the ˙CH_3,_ and ˙CF_3_ can act as a nucleophile and an electrophile, respectively, according to the generated MEP maps (see Fig. S3†). For ˙TF_3_⋯ and W–T–F_3_⋯˙CH_3_ complexes, the interaction energies increased in the same order as in Lewis base-containing complexes, confirming the potentiality of ˙CH_3_ to act as a Lewis base. For instance, the CCSD(T)/CBS interaction energies amounted to −1.11, −1.19, and −2.42 kcal mol^−1^ for H–Ge–F_3_⋯, ˙˙GeF_3_⋯, and F–Ge–F_3_⋯˙CH_3_ complexes, respectively. On the other hand, for ˙CF_3_-containing complexes, the interaction became stronger in the order F–T–F_3_⋯˙CF_3_ < ˙TF_3_⋯˙CF_3_ < H–T–F_3_⋯˙CF_3_, demonstrating that ˙CF_3_ could act as a Lewis acid. As an example, the CCSD(T)/CBS interaction energies were observed with values of −0.64, −0.85, and −0.97 kcal mol^−1^ for F–Ge–F_3_⋯, ˙GeF_3_⋯, and H–Ge–F_3_⋯˙CF_3_ complexes, respectively (see Table 2).

Overall, these results divulge that the strength of ^±^σ-hole interactions is higher compared to the ^±^R˙-hole ones, and the most stable complexes were observed for the ones containing Lewis bases. Besides, the strength of ^±^σ-hole and ^±^R˙-hole interactions in ˙TF_3_⋯ and W–T–F_3_⋯B/R˙/A complexes is not exclusive to the repulsive/attractive electrostatic interaction between the electrophilic site on tetrel atom and B/R˙/A, but also the interactions with the F_3_ atoms. These appealing findings seem to be consistent with other previous reports concerning the interactions of tetrel-containing molecules with Lewis bases, acids, and free radicals.^28,30,36,74,77,78^

#### 3.3.2. SAPT-EDA calculation

To elucidate the nature of ^±^σ-hole and ^±^R˙-hole interactions, symmetry adapted perturbation theory-based energy decomposition analysis (SAPT-EDA) was applied. SAPT-EDA partitions the interaction energy into four components; electrostatics (*E*_elst_), exchange (*E*_exch_), induction (*E*_ind_), and dispersion (*E*_disp_). These components and the total SAPT0 interaction energy for the ˙TF_3_⋯ and W–T–F_3_⋯B/R˙/A complexes were estimated and presented in Table 3.

The estimated SAPT0 interaction energy and its components (in kcal mol^−1^) for the ˙TF_3_⋯ and W–T–F_3_⋯B/R˙/A complexes (where T = C, Si, and Ge, W = H and F, B = Lewis bases, R˙ = free radicals, and A = Lewis acids)

**Table:** 

Complex	˙TF_3_/W–T–F_3_⋯B systems									
	NCH					FH				
	*E*_elst_	*E*_exch_	*E*_ind_	*E*_disp_	*E* ^SAPT0^_int_	*E*_elst_	*E*_exch_	*E*_ind_	*E*_disp_	*E* ^SAPT0^_int_
˙CF_3_⋯B	−1.26	1.77	−0.22	−1.63	−1.34	−0.54	0.81	−0.11	−0.98	−0.82
H–C–F_3_⋯B	0.22	0.92	−0.14	−1.19	−0.19	0.30	0.48	−0.08	−0.76	−0.04
F–C–F_3_⋯B	−1.37	1.58	−0.21	−1.57	−1.55	−0.67	0.77	−0.11	−0.97	−0.98
˙SiF_3_⋯B	−3.50	3.76	−0.67	−2.42	−2.83	−1.04	1.10	−0.21	−1.11	−1.26
H–Si–F_3_⋯B	−3.35	3.65	−0.64	−2.39	−2.72	−1.01	1.12	−0.21	−1.13	−1.23
F–Si–F_3_⋯B	−6.13	5.66	−1.24	−2.97	−4.68	−2.04	1.51	−0.35	−1.29	−2.17
˙GeF_3_⋯B	−6.12	7.20	−1.56	−3.63	−4.11	−1.22	1.71	−0.32	−1.44	−1.28
H–Ge–F_3_⋯B	−5.16	6.42	−1.26	−3.42	−3.41	−0.84	1.37	−0.25	−1.30	−1.02
F–Ge–F_3_⋯B	−24.0	28.45	−12.1	−7.75	−15.4	−3.28	2.57	−0.72	−1.76	−3.19

a Optimized structure could not be obtained, so no SAPT calculations were performed.

**Table:** 

Complex	˙TF_3_/W–T–F_3_⋯R˙ systems									
	˙CH_3_					˙CF_3_				
	*E*_elst_	*E*_exch_	*E*_ind_	*E*_disp_	*E* ^SAPT0^_int_	*E*_elst_	*E*_exch_	*E*_ind_	*E*_disp_	*E* ^SAPT0^_int_
˙CF_3_⋯R˙	−0.66	1.12	−0.06	−1.01	−0.61	−0.27	1.07	−0.08	−1.17	−0.45
H–C–F_3_⋯R˙	−0.36	0.93	−0.06	−0.95	−0.44	−0.86	1.46	−0.12	−1.41	−0.94
F–C–F_3_⋯R˙	−0.72	1.18	−0.06	−1.05	−0.66	−0.25	1.25	−0.10	−1.30	−0.41
˙SiF_3_⋯R˙	−1.43	2.07	−0.18	−1.46	−1.01	−0.33	1.48	−0.17	−1.46	−0.46
H–Si–F_3_⋯R˙	−1.53	2.25	−0.20	−1.55	−1.03	−0.42	1.72	−0.20	−1.58	−0.48
F–Si–F_3_⋯R˙	−2.33	3.11	−0.39	−1.82	−1.43	0.03	1.91	−0.29	−1.65	−0.06
˙GeF_3_⋯R˙	−2.31	3.65	−0.40	−2.20	−1.26	−1.01	3.21	−0.42	−2.38	−0.60
H–Ge–F_3_⋯R˙	−2.02	3.26	−0.32	−2.07	−1.15	−1.05	2.96	−0.36	−2.28	−0.73
F–Ge–F_3_⋯R˙	−4.68	6.60	−1.47	−3.01	−2.57	−0.20	3.56	−0.75	−2.46	0.14

**Table:** 

Complex	˙TF_3_/W–T–F_3_⋯A systems									
	HCN					HF				
	*E*_elst_	*E*_exch_	*E*_ind_	*E*_disp_	*E* ^SAPT0^_int_	*E*_elst_	*E*_exch_	*E*_ind_	*E*_disp_	*E* ^SAPT0^_int_
˙CF_3_⋯A	0.65	0.45	−0.24	−0.81	0.05	0.40	0.03	−0.09	−0.21	0.13
H–C–F_3_⋯A	−0.60	0.81	−0.35	−1.06	−1.21	−0.12	0.39	−0.33	−0.66	−0.72
F–C–F_3_⋯A	0.82	0.44	−0.25	−0.83	0.19	0.75	0.08	−0.15	−0.34	0.35
˙SiF_3_⋯A	0.36	0.10	−0.14	−0.44	−0.11	0.29	0.01	−0.06	−0.14	0.10
H–Si–F_3_⋯A	0.43	0.14	−0.16	−0.51	−0.10	0.02	0.01	−0.03	−0.06	−0.06
F–Si–F_3_⋯A	1.18	0.07	−0.13	−0.39	0.74	—a	—a	—a	—a	—a
˙GeF_3_⋯A	0.41	0.49	−0.30	−0.91	−0.31	0.53	0.08	−0.16	−0.34	0.10
H–Ge–F_3_⋯A	0.23	0.56	−0.33	−0.97	−0.51	0.21	0.05	−0.13	−0.29	−0.16
F–Ge–F_3_⋯A	1.03	0.03	−0.08	−0.27	0.67	—a	—a	—a	—a	—a

A perusal of the data in Table 3 shows that for ^−^σ-hole and ^−^R˙-hole interactions, the *E*_disp_, *E*_elst_, and *E*_ind_ terms are negative, unlikely; the *E*_exch_ is positive. As well, all these terms increased in the order C < Si < Ge. Generally, it is apparent that the *E*_disp_ governs the ^−^σ-hole and ^−^R˙-hole interactions, followed by the *E*_elst_, and *E*_ind_. For instance, *E*_disp_ contributions were 89, 90, and 69% out of the total attractive terms for the H–C–F_3_⋯NCH, ⋯FH, and ⋯˙CH_3_ complexes, respectively. On the other hand, for Ge-containing molecules interacting with NCH as Lewis base, the interactions were dominated by *E*_elst_; where the *E*_elst_ contribution to the total interaction energy ranged from −5.16 to −24.0 kcal mol^−1^ and the *E*_disp_ contribution ranged from −3.42 to −7.75 kcal mol^−1^ (see Table 3). These findings demonstrated the importance of electrostatic and dispersion energies in stabilizing ˙TF_3_⋯ and W–T–F3⋯NCH/FH/˙CH_3_ complexes.

Turning now to ^+^σ-hole and ^+^R˙-hole interactions, the *E*_disp_ term was the most important attractive contributor. Besides, generally, the *E*_elst_ and *E*_ind_ terms help in promoting the strength of interactions for ˙TF_3_/W–T–F_3_⋯˙CF_3_ and ⋯HCN/HF complexes, respectively. For example, the *E*_disp_, *E*_elst_, and *E*_ind_ were −2.38, −1.01, and −0.42 kcal mol^−1^ for ˙GeF_3_⋯˙CF_3_ complex, respectively (see Table 3).

Taken together, these results disclose that the stability of the studied complexes-containing Lewis bases, Lewis acids, and radicals are attributed mainly to the dispersion forces, with enhanced contributions from electrostatic and induction forces.

#### 3.3.3. RDG–NCI analysis

**Fig. 5:**
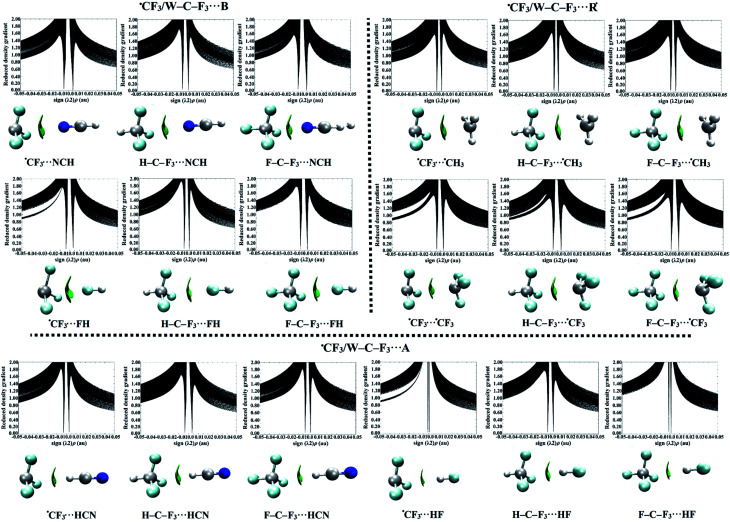
NCI isosurfaces of the studied ˙CF_3_⋯ and W–C–F_3_⋯B/R˙/A complexes (where W = H and F, B = Lewis bases, R˙ = free radicals, and A = Lewis acids). NCI isosurfaces are colored on a blue-green-red (BGR) scale with blue and red for attractive and repulsive interactions, respectively. The corresponding sign (*λ*_2_)*ρ vs.* RDG plots to the NCI isosurfaces are also depicted.

To reveal the characteristics and nature of ^±^σ-hole and ^±^R˙-hole interactions, NCI calculations were carried out for the optimized ˙TF_3_⋯ and W–T–F_3_⋯B/R˙/A complexes. NCI isosurfaces and plots of the reduced density gradient (RDG) *versus* the electron density (*ρ*) multiplied by the sign of the second Hessian eigenvalue (*λ*_2_) of the analyzed complexes were generated at MP2/aug-cc-pVTZ (PP) level of theory and presented in Fig. S4 and S5,† respectively. The RDG–NCI plots of carbon-containing complexes were represented in Fig. 5, as an example.

The results obtained from the NCI isosurfaces demonstrated the favorable ^−^σ-hole and ^−^R˙-hole interactions, showing disc-shaped green isosurfaces between the interacting fragments (Fig. S4†). Besides, the green isosurfaces became larger with increasing interaction strength, converting to blue ones for the strong interactions as observed in the F–Ge–F_3_⋯NCH complex. Moreover, the corresponding spikes of sign (*λ*_2_)*ρ* at low densities confirmed the existence of such attractive interactions (sign (*λ*_2_)*ρ* < 0). The location of spikes had a greater deviation from zero, and its shape became broader with enhancing the interaction energy (see Fig. S5†).

For ^+^σ-hole and ^+^R˙-hole interactions, disc-shaped green isosurfaces were also noticed for the studied complexes, asserting the existence of such interactions (see Fig. S4†). Furthermore, green isosurfaces were observed between the F_3_ atoms and Lewis acids, revealing their role in stabilizing the Lewis acids-containing complexes. The favorable interactions were also characterized by spikes at the negative sign (*λ*_2_)*ρ*. For instance, the spikes corresponding to each of H–C–F_3_⋯HCN and ⋯˙CF_3_ complexes were located at negative sign (*λ*_2_)*ρ*, indicating the probability of such interactions to dwell (see Fig. S5†).

In summary, these findings underlined that the ^−^σ-hole and ^−^R˙-hole interactions are the most favorable ones, where the large green isosurfaces and broad spikes were observed in their complexes.

## 4. Conclusion

The potentiality of the tetrel-containing molecules to engage in interactions with Lewis bases, free radicals, and Lewis acids along the R˙-hole extension, forming ^±^R˙-hole interactions, was uncovered for the first time. The characteristics of ^±^R˙-hole interactions were compared to the ^±^σ-hole analogs in ˙TF_3_⋯ and W–T–F_3_⋯B/R˙/A complexes, respectively. The nature and characteristics of such interactions were discussed adequately by interpreting the MEP, *V*_s,max_, PoC, interaction energy, SAPT, and NCI-based results. These findings suggested that (i) the occurrence of σ-hole and R˙-hole over the tetrel atom was conspicuous, (ii) utilizing the PoC approach, the ability of the ˙TF_3_⋯ and W–T–F_3_ monomers to participate in favorable electrostatic interaction with Lewis bases and acids was demonstrated, (iii) the highest molecular stabilization energies were obtained in case of ±1.00 au PoC due to the effectual role of polarization, (iv) ˙TF_3_ and W–T–F_3_ monomers showed more favorable interactions with Lewis bases than Lewis acids, (v) a comparison of the ^±^σ-hole and ^±^R˙-hole interactions revealed the former is more energetically favorable, (vi) the strength of such interactions is not exclusive to the repulsive/attractive electrostatic interaction between the electrophilic site on tetrel atom and B/R˙/A, but also the interactions with the F_3_ atoms, (vii) generally, the dispersion force played a critical role in stabilizing the tetrel-containing complexes, with a non-neglectable contribution of the electrostatic and induction forces to the interactions with Lewis bases and acids, respectively, and (viii) disc-shaped green isosurfaces were noticed between the interacting fragments, providing evidence of the presence of ^±^σ-hole and ^±^R˙-hole interactions. Thus, the upshot of these results adds to the growing body of research and will also be advantageous to the ones related to the crystal engineering and materials science fields.

## Conflicts of interest

There are no conflicts to declare.

## Supplementary Material

RA-011-D0RA09564H-s001
