# Supplementary material for: Comparison of ±σ-hole and ±R˙-hole interactions formed by tetrel-containing complexes: a computational study
Source: RSC Adv. 2021 Jan 19;11(7):4011–21. doi: 10.1039/d0ra09564h (PMC8694216; doi:10.1039/d0ra09564h)
Supplement: RA-011-D0RA09564H-s001 [file RA-011-D0RA09564H-s001.pdf]

Electronic Supplementary Material (ESI) for RSC Advances.
This journal is © The Royal Society of Chemistry 2021

Comparison of $^{\pm}\sigma$-hole and $^{\pm}R^{\bullet}$-hole interactions formed by tetrel-containing complexes: A computational study

Mahmoud A. A. Ibrahim[1,*], and Ebtisam M. Z. Telb[1]

[1]Computational Chemistry Laboratory, Chemistry Department, Faculty of Science, Minia University, Minia 61519, Egypt

* Corresponding author: Mahmoud A. A. Ibrahim; m.ibrahim@compchem.net

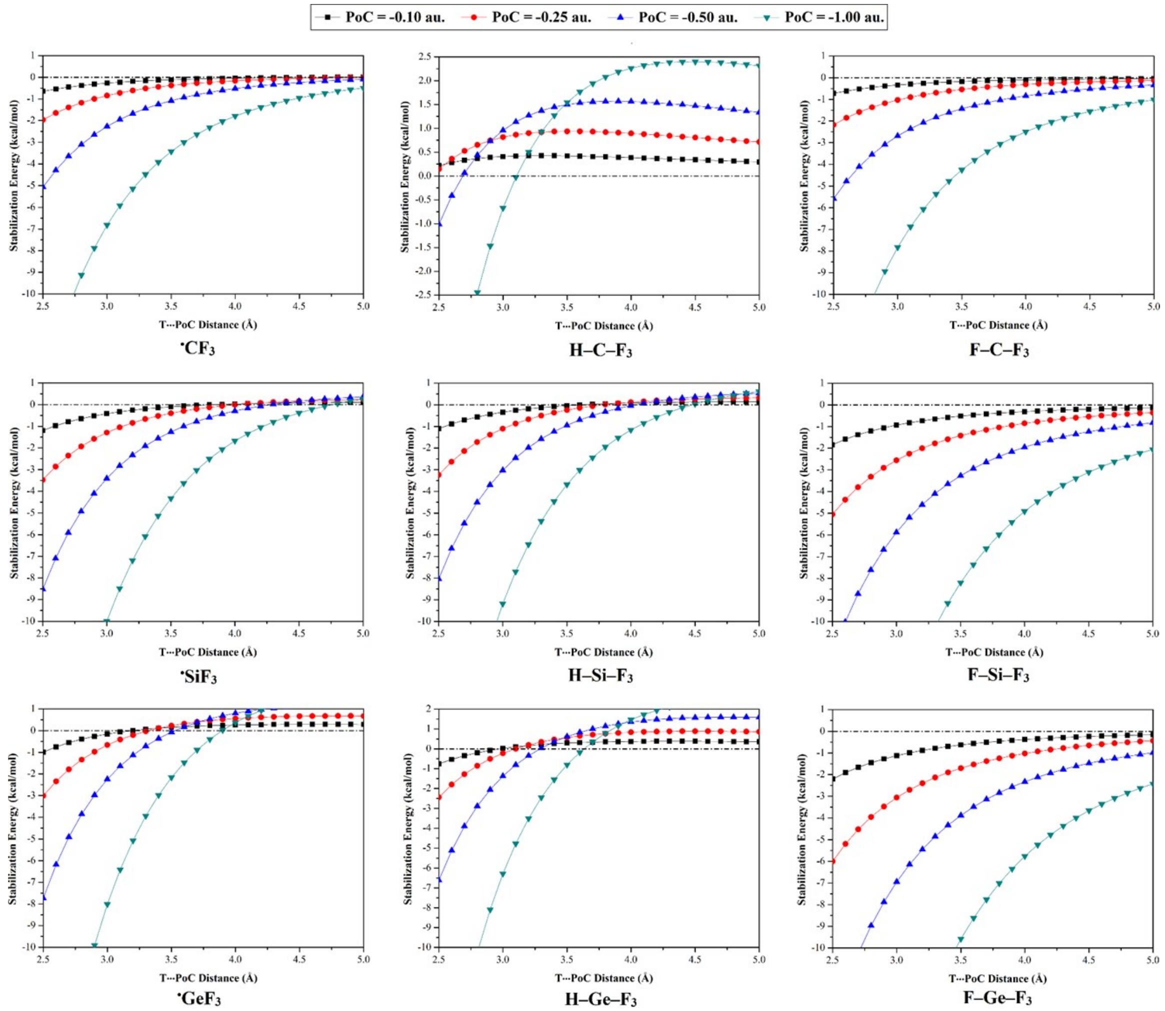

Figure S1: Generated molecular stabilization energy curves for the $^{\bullet}TF_3$ and W–T–F_3 systems (where T = C, Si, and Ge, and W = H and F) in the presence of −0.10, −0.25, −0.50, and −1.00 au PoCs at T···PoC distances ranging from 2.5 Å to 5.0 Å.

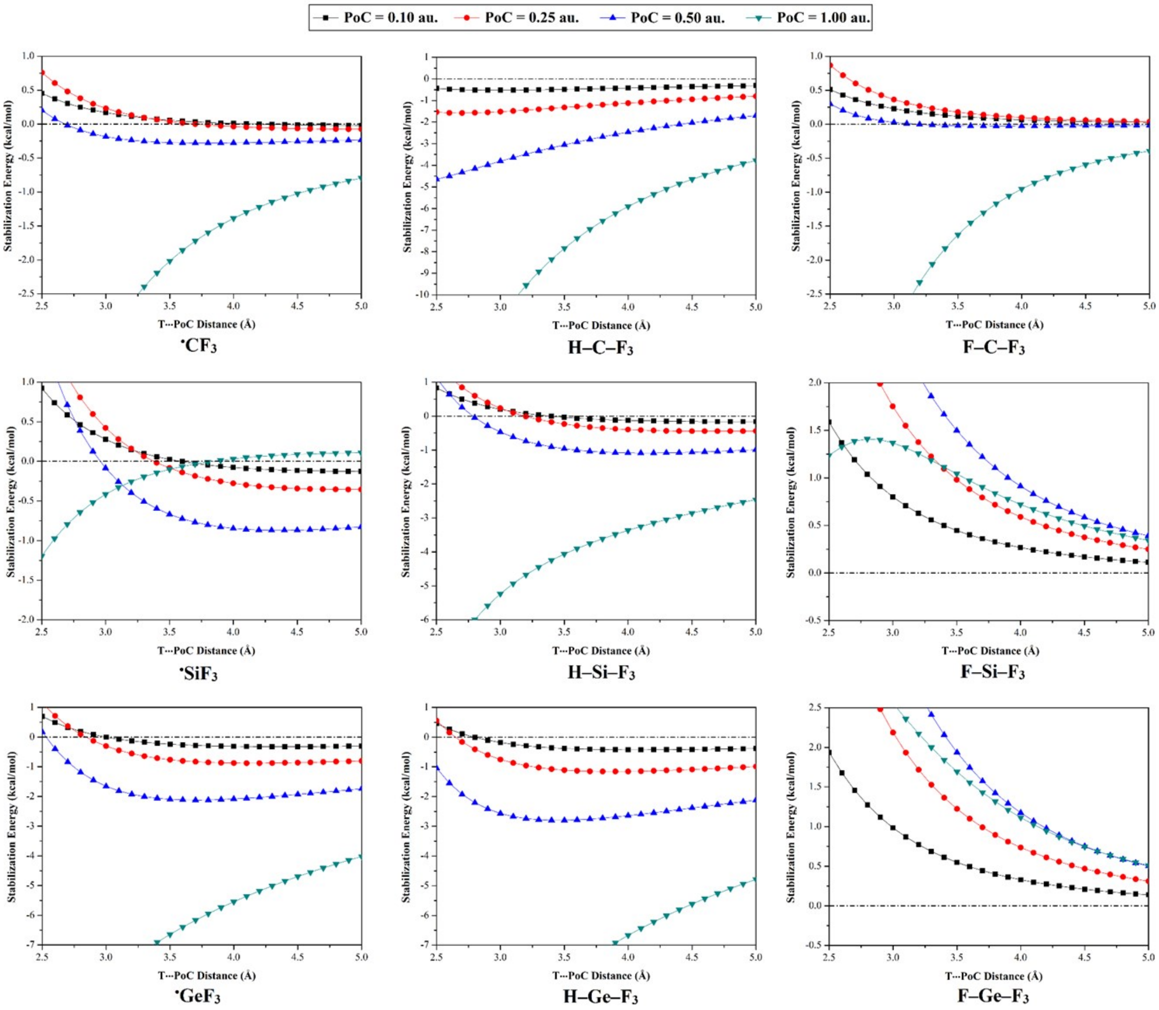

Figure S2: Generated molecular stabilization energy curves for the $^{\bullet}TF_3$ and W–T–F_3 systems (where T = C, Si, and Ge, and W = H and F) in the presence of 0.10, 0.25, 0.50, and 1.00 au PoCs at T···PoC distances ranging from 2.5 Å to 5.0 Å.

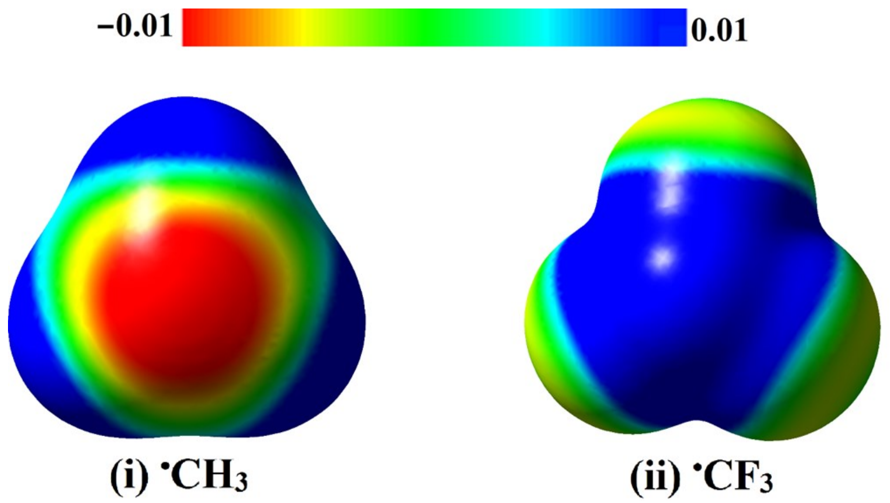

Figure S3: The generated molecular electrostatic potential (MEP) maps of the (i) $^{\bullet}CH_3$ and (ii) $^{\bullet}CF_3$ molecules at the MP2/aug-cc-pVTZ level of theory. The MEP maps were plotted at 0.002 au electron density contours, and the electrostatic potentials vary from −0.01 au (red) to 0.01 au (blue).

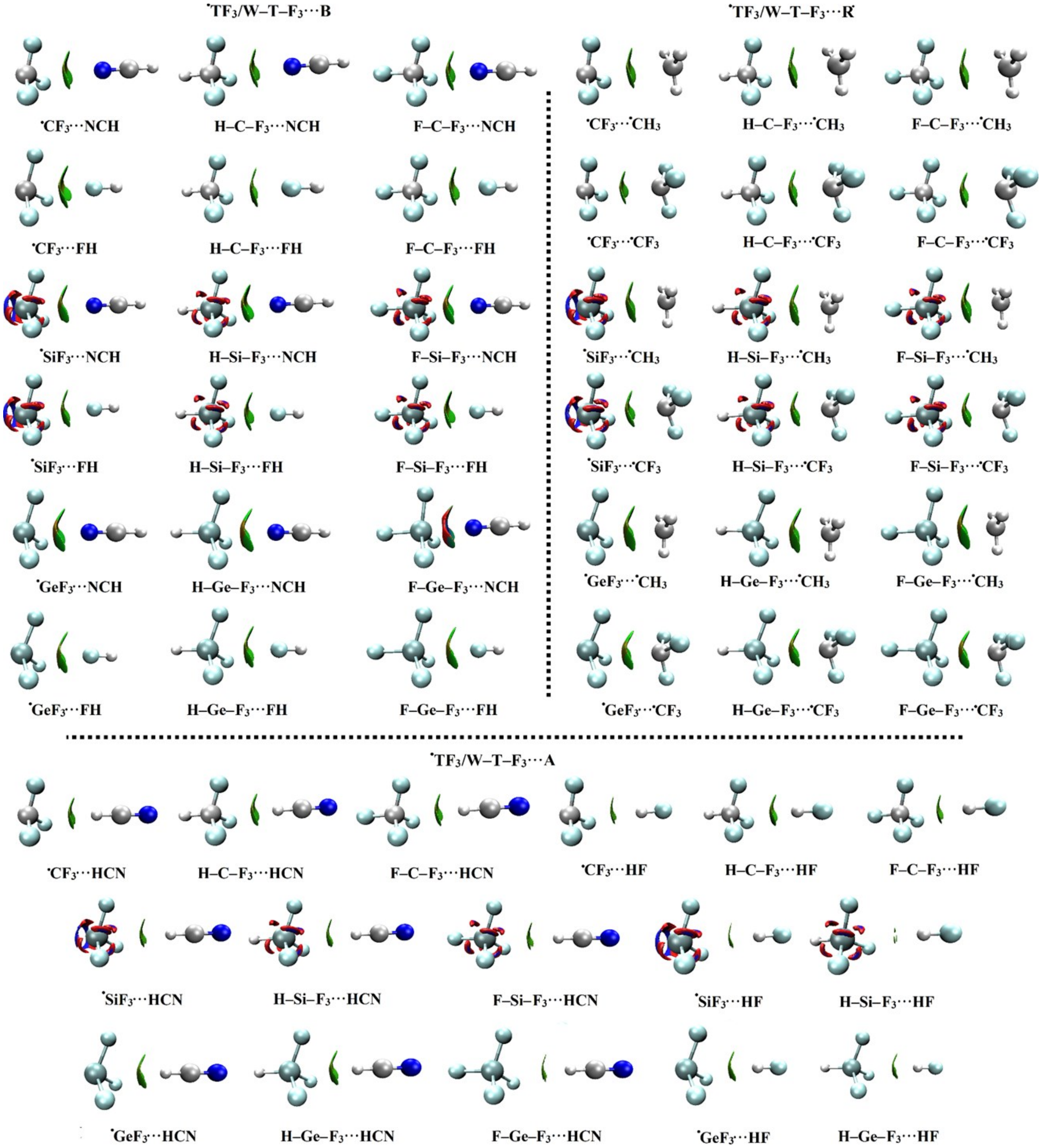

Figure S4: NCI isosurfaces for the studied $^{\bullet}TF_3\cdots$ and W–T–$F_3\cdots$ B/R$^{\bullet}$/A complexes (where T = C, Si, and Ge, W = H and F, B = Lewis bases, R$^{\bullet}$ = free radicals, and A = Lewis acids).

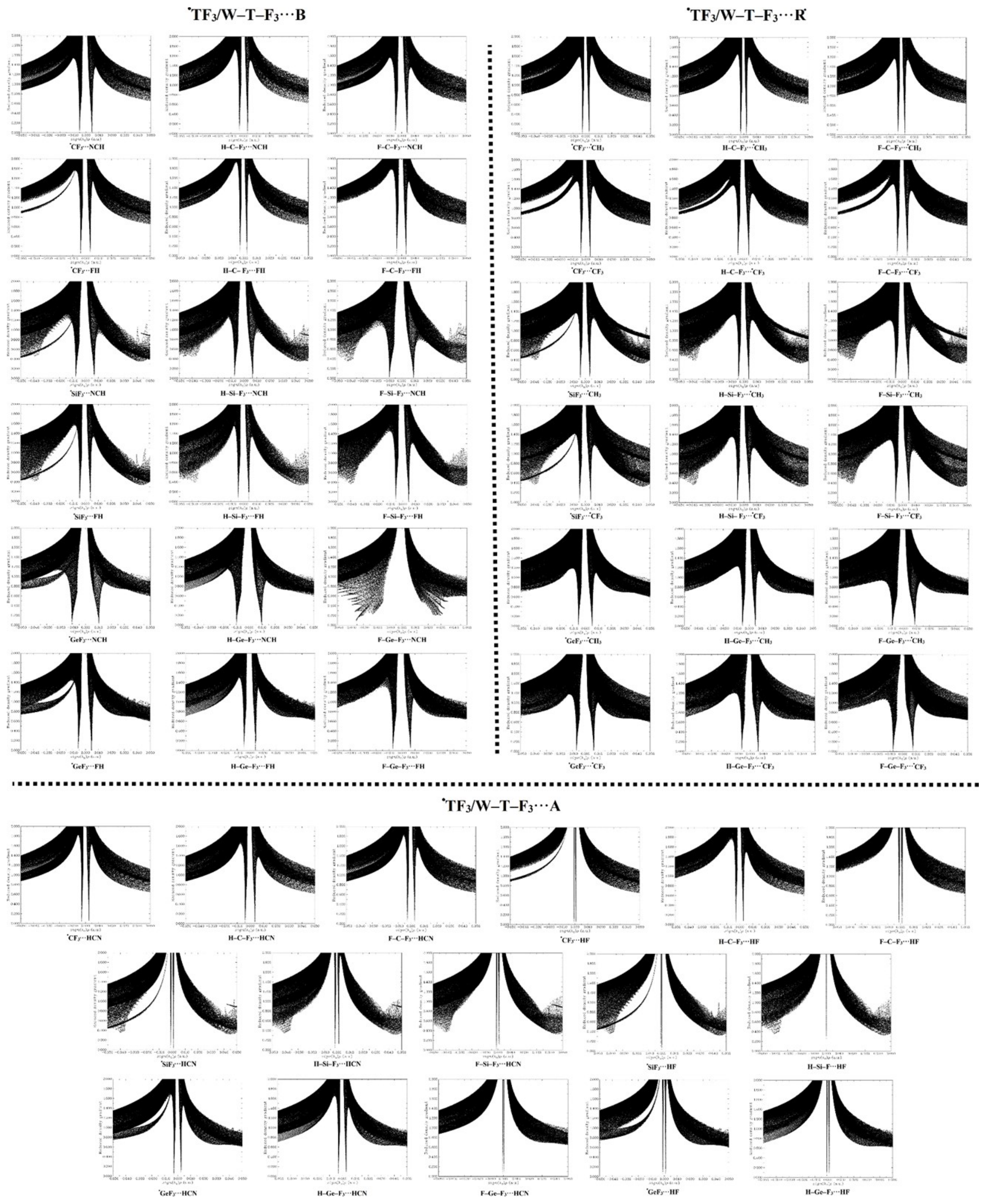

Figure S5: Plots of the electron density and its reduced gradient for the studied •TF$_3$··· and W–T–F$_3$··· B/ R•/A complexes (where T = C, Si, and Ge, W = H and F, B = Lewis bases, R• = free radicals, and A = Lewis acids).
